# Thyroid Hormone Enhances Neurite Outgrowth in Neuroscreen 1 Cells

**DOI:** 10.31531/2581-4745.1000104

**Published:** 2018-03-30

**Authors:** Oldham CE, Wooten CJ, Williams AB, Dixon S, Lopez D

**Affiliations:** Department of Pharmaceutical Sciences, Biomanufacturing Research Institute and Technology Enterprise (BRITE), College of Arts and Sciences, North Carolina Central University, Durham, USA

**Keywords:** Alzheimer’s disease, NS-1 cell, Thyroid hormones, Amyloid precursor protein, Tau, Exon 10 splicing

## Abstract

**Objectives:**

Alzheimer’s disease (AD) is a neurodegenerative disorder that affects millions of individuals. Moreover, hypothyroidism has been identified as one of the risk factors that may contribute to the development of AD. Here, we investigated whether there was a correlation among expression levels of proteins involved in the formation of AD lesions, neurite outgrowth, and thyroid hormone levels.

**Methods:**

Cells were grown in media supplemented with different levels of 3,5,3’-triiodothyronine (T3) and then processed for neurite outgrowth and to prepare RNA samples. RNA samples were analysed using quantitative real-time PCR. Protein levels were measured using in cell-Western blotting analysis.

**Results:**

By using neurite outgrowth studies, it was demonstrated that T3 treatment enhanced neurite outgrowth in NS-1 cells in a time- and dose-dependent manner. Quantitative real-time PCR studies further confirmed that NS-1 cells expressed substantial levels of TRα and significantly less TRβ, either of which could be responsible for the T3-dependent effects on neurite outgrowth. Although the overall tau protein expression was not affected in response to T3 treatment, the splicing of tau exon 10 was impacted in the direction of producing more tau molecules that excluded the exon (tau 3R).

**Conclusion:**

The results of this study are critical not only to understand the probable link between hypothyroidism and AD but also in providing the basis for future prevention and treatment of AD in hypothyroid patients.

## Introduction

Alzheimer’s disease (AD) is a progressive neurodegenerative disorder that currently affects more than 25 million individuals, a number that is expected to double by the year 2040 [1,2]. Although the course of the disease is unique for every person, several psychological and physical symptoms are characteristic of all patients [3,4]. The earliest and most prominent symptom of AD is a loss of short-term memory [3,4]. Other symptoms such as language impairment, difficulties completing complex tasks, depression, psychotic episodes, and behavioural changes also occur as the disorder progresses [3,4]. These symptoms exist as the manifestation of the main lesions that can be found in the patients with AD which consist of extracellular amyloid or senile plaques (SPs) and intracellular neurofibrillary tangles (NFTs) [5,6]. SPs are made of aggregations of the short peptide (39–43 amino acids) known as amyloid beta (Aβ) which results from abnormal processing of amyloid precursor protein (APP) [7,8]. APP is a transmembrane protein considered to be critical to neuron growth, survival, and post-injury repair [7,8]. AP is made when APP is sequentially cleaved by two proteases, β-secretase (also called β-site APP-cleaving enzyme 1 or BACE1) and γ-secretase [5]. Upon its production, Aβ is released into the extracellular space where it can be assembled into different conformations [5]. Although Aβ monomers are soluble and harmless when present at sufficiently high concentrations, they can form beta-sheet-rich tertiary structures that aggregate into amyloid plaques outside neurons [9,10]. While Aβ accumulates in SPs of AD patients, microtubule-associated protein tau accumulates in NFTs [11,12]. The proper function of tau, when phosphorylated, is to stabilize microtubules in the cytoskeleton of neurons [11,12]. In AD patients, tau becomes abnormally hyperphosphorylated leading to the production of impaired helical filaments that aggregate to form NFTs inside nerve cell bodies [11,12]. The formation of the NFTs causes disintegration of microtubules and results in the dysfunction of the axonal transport [11,12].

Risk factors described for AD include aging, familial aggregation for AD, familial aggregation for Down’s syndrome and Parkinson’s disease, late maternal age, head trauma, history of depression, diabetes mellitus, hypertension, obesity, smoking, cognitive or physical inactivity, and history of hypothyroidism [12–17]. Hypothyroidism, which results from decreased production of thyroid hormones (3,5,3’-triiodothyronine or T3 and thyroxine or T4) due to the destruction of the thyroid tissue is the second most common endocrine disorder in the United States [18]. Similarly, hypothyroidism also becomes more common with aging as with AD [19]. Interestingly, among the symptoms associated with untreated hypothyroidism include memory loss, lack of concentration, and dementia [19–21], which mimic some of the initial symptoms of AD [3,4]. Therefore, it is possible that the overlap between some of the symptoms of these two diseases contributes to the susceptibility to AD for patients who have been diagnosed with hypothyroidism. Several studies have shown that T3 is essential for healthy brain development, maturation, and function [22–26]. Studies using embryonic and adult mice demonstrated that T3 plays a vital role in the development and maintenance of basal forebrain cholinergic neurons which are typically altered by AD [26–28]. A link between hypothyroidism and AD susceptibility has been suggested before. The current study focused on determining whether T3 affects neurite outgrowth and the expression levels of proteins involved in the formation of AD lesions in rat NS-1 cells. Initial results suggested that T3 enhanced neurite outgrowth and further potentiates the effects of nerve growth factor (NGF), which is essential for the healthy growth, maintenance, and survival of neuron cells.

## Materials and Methods

The rat neuronal-like NeuroScreen 1 (NS-1) and rat hepatic H4IIE cell lines were from American Type Culture Collection (ATCC; Manassas, VA). RPMI 1640 medium, low glucose Dulbecco’s modified Eagle’s medium (LG-DMEM), antibiotic (penicillin/streptomycin) solution, phosphate-buffered saline (PBS), L-glutamine, activated charcoal, Dowex (AG-1X-10) resin, Ponceau S Stain, Cell Viability Green Indicator dye, reverse transcriptase system, fetal bovine serum (FBS), and the SYBR Green PCR Master Mix were purchased from Thermo Scientific (Logan, UT). NGF was from Fisher Scientific (Pittsburg, PA). 3,5,3’-Triiodothyronine (T3) was from Sigma–Aldrich (St. Louis, MO). The free T3 ELISA kit was obtained from Fitzgerald (Acton, MA). TRI Reagent was purchased from Molecular Research Center (Cincinnati, OH). The Turbo DNA-free kit was acquired from Ambion (Austin, TX). Rabbit anti-TRα/β (FL-408; diluted 1:100), goat anti-amyloid A4 (diluted 1:100), and goat anti-actin (diluted 1:250) specific antibodies were obtained from Santa Cruz Biotechnology (Santa Cruz, CA). Rabbit anti-Aβ (diluted 1:100), sheep anti-tau (diluted 1:333), mouse anti-actin (diluted 1:500), anti-rabbit Dylight 680, anti-mouse Dylight 680, anti-sheep Dylight 680, and anti-goat Dylight 800 were from Pierce Thermo Scientific Pierce (Rockford, IL). All other materials used were from Fisher Scientific or Sigma–Aldrich.

### Preparation of thyroid hormone deficient medium

Thyroid hormone deficient serum (THD) was prepared mainly as previously described [29]. Stripped serum was tested for cholesterol and total T3 levels. Cholesterol levels were only slightly decreased from 15.17 mg/dL to 13.1 mg/dL after the processing. Total T3 levels were reduced from 1.996 ng/mL to undetectable levels after the processing. This method also removes T4 levels [29].

### Cell culture

NS-1 cells were maintained at a density of 10^6^ cells per 75-cm^3^ flask in RPMI 1640 medium containing 10% FBS, 2 mM L-glutamine, and antibiotics, at 37^o^C under a humidified atmosphere and 5% carbon dioxide. NS-1 is a neuronal-like cell line sub-cloned from the rat adrenal pheochromocytoma PC12 cell line [30]. The latter has been used as a standard model system for neurons [30]. It is important to mention that the NS-1 cells exhibit several advantages over the PC12 cells that make them a better model for neuronal studies [30]. First, NS-1 cells grow 50–80% faster and with less cell aggregation than the PC12 cells [30]. Secondly, NS-1 cells have a high and accelerated response to NGF (measurable neurites within two days) as compared to the PC12 cells [30]. For most of the experiments, the NS-1 cells were seeded in six-well plates at the density of 2 ×10^4^ viable cells per well in RPMI 1640 medium supplemented as indicated above. After 24 h, the medium was replaced with one of the following experimental media: 1) maintaining medium (described above; 10% FBS medium), 2) a medium containing 1% FBS instead of 10% (1% FBS medium), or 3) a medium containing 1% THD instead of FBS. Treatments, which included NGF (50 ng/ml) or T3 (increasing amounts from 1 up to 10 nM), were added to the cells 24 h after changing the medium to one of the experimental media. Incubations with the treatments were carried out for 24–72 h as described in the figure legends. For thyroid hormone receptor (TR) expression studies, rat hepatic H4IIE cells were cultured as described for the NS-1 cells, but in LG-DMEM medium supplemented with 10% FBS and antibiotics and used in the preparation of RNA or in-cell Western assays. For some experiments, cell viability was measured using a cell-permeable viability green indicator dye using the manufacturer’s instructions. The green fluorescence produced by the effects of the live cells on the viability indicator dye was detected using a BMG LabTech PHERA star TM5 fluorescence plate reader (ex/em: 495/515 nm).

### Analysis of neurite outgrowth

After treating as indicated above, cell images were acquired using the inverted phase-contrast microscope IX51 (Olympus Corp, Waltham, MA) and analyzed using the NIS Elements BR 3.0 Imaging software (Nikon Inc, Melville, NY). Cells from each treatment group were examined in a minimum of three random non-overlapping fields per well. Cells containing at least one neurite with a length >20 μm or at least twice the diameter of the cell were counted as cells with neurites and expressed as a percent of the total cells. Data were presented as a percentage of cells with neurites.

### RNA preparation and quantitative real-time polymerase chain reaction (qRT-PCR)

Total RNA was prepared using the acid guanidinium thiocyanate-phenol-chloroform extraction method [31] which employs TRI Reagent. The concentration of the RNA samples and the purity of the RNA preparation were determined using a Nanodrop 2000. The integrity of the RNA was also confirmed using electrophoresis. DNase I treatment and reverse transcriptase reactions were carried out using standard methods. qRT-PCR reactions were performed using 100 ng of ssDNA, the Applied Biosystems SYBR Green PCR Master Mix, and the AB real-time PCR system. Rat TRα and TRβ specific primers were obtained from SA Biosciences (Frederick, MD).

The sizes of the TRα and TRβ fragments that were amplified using these primers were 86 and 117 bp, respectively. Rat 18s rRNA (5’-GTAACCCGTTGAACCCCATT-3’ and 5’-CCATCCAATCGGTAGTAG CG-3’), rat APP (5’-AGAAGTGAAGATGGATGCGG-3’ and 5’-TTGCTATGACAACGCCACC-3’), and rat tau (5’-TGGAGAGAAGAGAGAGTGAGAG-3’ and 5’-TGGTCAGCCTGTCTATGAGG-3’) specific primers were synthesized by Eurofins MWG Operon (Huntsville, AL). In some experiments, primers specific for rat tau 3R (5’-ACTGAGAACCTGAAGCACCA-3’ and 5’-TTGCTCAGGTCCACTGGCTTGTA-3’) and rat tau 4R (5’-GCAGATAATTAATAAGAAGCTGGA-3’ and 5’-GTGTTTGATATTGTCCTTTGAGC-3’), also synthesized by Eurofins, were employed.

The parameters for the PCRs were: denaturation at 95^o^C for 10 min, followed by 45 cycles of denaturation at 95^o^C for 30 sec, annealing for 15 sec, and extension at 72^o^C for 30 sec. The annealing temperatures used were 60^o^C for TRα, TRβ, 18s rRNA, and APP, and 63^o^C for all three sets of tau primers. Calculations were carried out using the comparative Ct method.

### In-cell western analysis

NS-1 (and H4IIE in the case of TR-proteins) cells were cultured and treated with T3 as indicated above in clear 96-well plates. After treatments, media were removed, and the cells were fixed in 3.5% formaldehyde/1X PBS for 20 min at room temperature. Cells were then permeabilized by washing 5 times, 5 min per wash, with 1X PBS/0.1% Triton X-100 and blocked in Rockland blocking buffer (Gilbertsville, PA) for 1.5 h at room temperature. Incubation with primary antibodies, diluted in blocking buffer/0.1% Tween 20 (dilution factors have been included above), was carried out overnight at 4^o^C. An actin-specific antibody (goat or mouse; depending on the species of the other antibody) was used as the internal control for this experiment and was incubated for 2.5 h at room temperature. Washing after the primary antibodies was done with 1X PBS/0.1% Tween 20 for a total of 5 times, once again, 5 min per wash. Infrared anti-species Dylight 680 or Dylight 800 secondary antibodies (depending on the primary antibodies) diluted 1:400 in PBS/0.5% Tween-20 was added to the wells and incubated for 1 hour at room temperature in the dark. Washing was carried out with 1X PBS/0.1% Tween 20 as described above. Plates were imaged on a Li-COR infrared scanner using microplate settings with a sensitivity of 5 and a resolution of 169 μm in both the 700 and 800 nm wavelength channels.

Data were acquired using the Li-COR software. TRs, AA4, Aβ, and tau values were background subtracted from wells treated with secondary antibody only and then normalized to the actin signal.

### Statistical analysis

Data from the individual parameters for at least three independent measurements (n=3) were compared using analysis of variance (ANOVA) followed by Student–Newman–Keuls multiple comparison tests or the Student’s t-test when applicable, using the GraphPad Prism 6 software (GraphPad Software, Inc., La Jolla, CA). A p<0.05 was considered significant for all tests.

## Results

**Figure 1:**
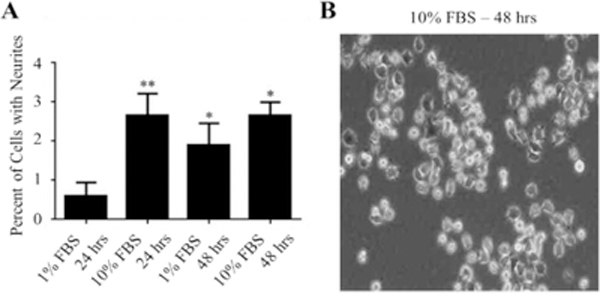
Effects of different media on neurite outgrowth. NS-1 cells were incubated in the indicated media for 24 and 48 h before microscope images were taken for neurite outgrowth evaluation. (A) Data are presented as a percentage mean of cells with neurites ± standard error of the mean (SEM) for n=15 for all the conditions. “*” (p<0.05) and “**” (p<0.01) were obtained by comparing to the 1% FBS 24 hrs group using ANOVA followed by Student–Newman–Keuls multiple comparison tests. (B) Morphology of the NS-1 cells after 48 h in 10% FBS medium. This experiment has been repeated for at least 5 times.

Initial studies were performed to examine the effects of 10% FBS and 1% FBS media on neurite outgrowth in NS-1 cells. The total number of cells and the number of cells containing neurites were counted and used to determine the percentage of cells with neurites. As shown in Figure 1A, the percentages of cells with neurites in both media, independently of the incubation time, were below 3%.

Maximum levels (2.7%) were obtained in the presence of 10% FBS medium, also regardless of the time examined (Figure 1A). Cells remained mainly undifferentiated, and any apparent neurite had a size less than one diameter of the size of the cell (Figure 1B).

**Figure 2:**
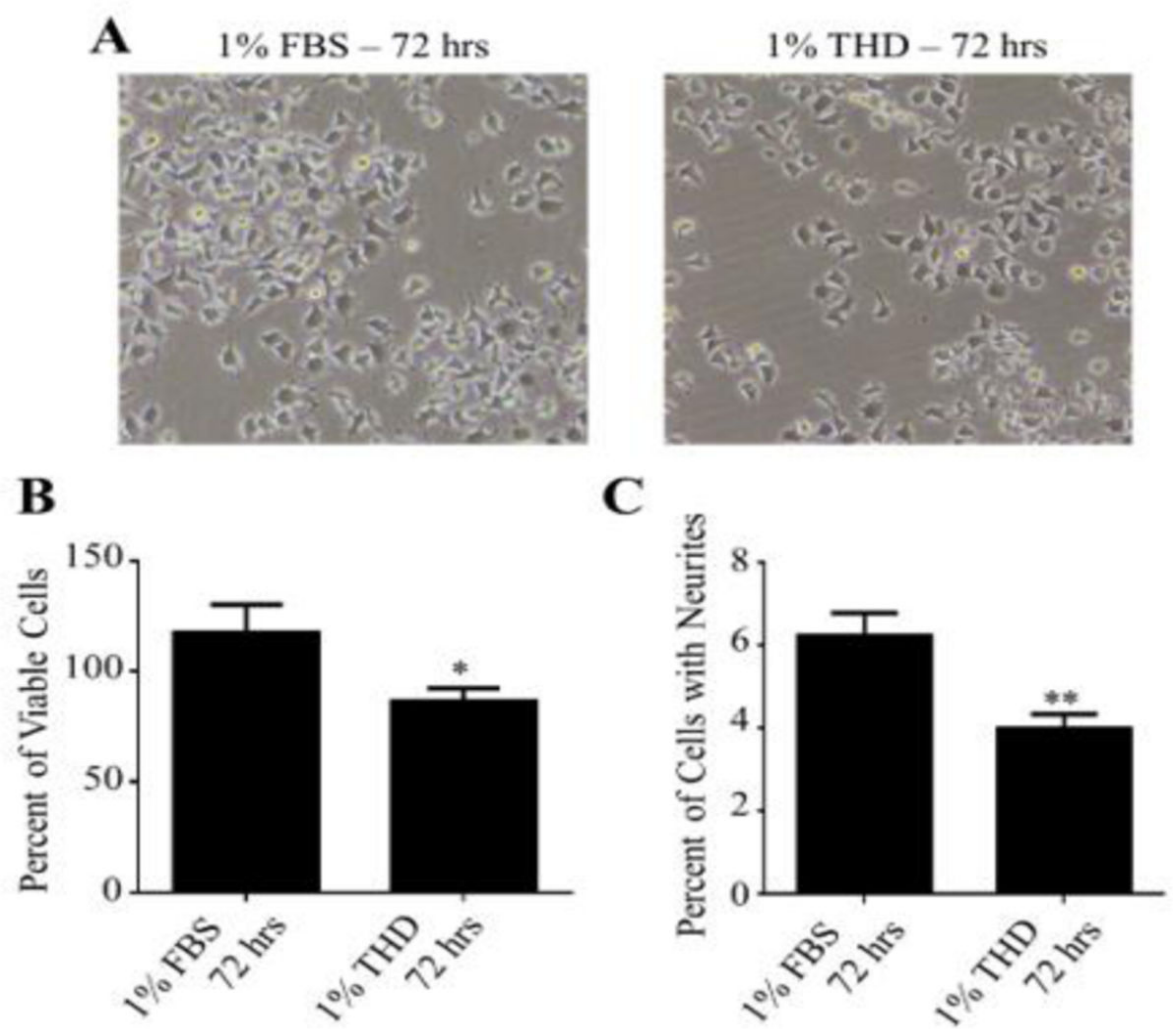
Effects of hypothyroidism on neurite outgrowth in NS-1 cells. Cells were incubated in the indicated media for 72 h before evaluation of neurite outgrowth. (A) Typical microscope images of cells exposed to the two media. These experiments were repeated at least 4 times. (B) Quantitated results from cell viability studies using nuclei green stain as described under Materials and Methods. The data are presented as a percentage mean of viable cells ± SEM (n=8 for 1% FBS; n=16 for 1% THD). (C) Data are presented as a percentage mean of cells with neurites ± SEM for n=15 for all the conditions. “*” (p<0.05) and “**” (p<0.01) were obtained by comparing the two groups using the Student’s t-test.

The next step was to measure the effects of a hypothyroid medium (THD) on neurite outgrowth. For this, cells were incubated for 72 h in 1% FBS or 1% THD medium before being analyzed for neurite outgrowth. Figure 2A illustrates representative fields of cells treated with 1% FBS or 1% THD medium for 72 h.

**Figure 3:**
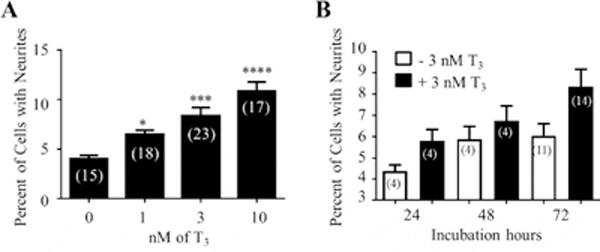
Effects of 3,5,3’-triiodothyronine (T3) on neurite outgrowth in NS-1 cells. (A) Dose-response studies. Cells were incubated in 1% THD media with increasing amounts (nM) of T3 for 72 h before evaluation of neurite outgrowth. The data are presented as a percentage mean of cells with neurites ± SEM. “*” (p<0.05), “***” (p<0.001) and “****” (p<0.0001) were obtained by comparing to the group without T3 using ANOVA followed by Student–Newman–Keuls multiple comparison tests. (B) Time course studies. Cells were incubated in 1% THD media ± 3 nM T3 for 24, 48, and 72 h before evaluation of neurite outgrowth. The data are presented as a percentage mean of cells with neurites ± SEM. “*” (p<0.05) was obtained by comparing to the 72 h group without T3 using the Student’s t-test. Numbers within parenthesis inside each bar correspond to the replicates (“n”) for each group.

Incubating in 1% THD significantly (p<0.05) reduced the viability of the NS-1 cells by 27% as compared to cells grown in 1% FBS (Figure 2B). As depicted in Figure 2C, changing the medium to 1% THD caused a significant decrease (36% reduction; p<0.01) in neurite outgrowth after 72 h. T3 dose-response studies were then carried out in NS-1 cells cultured in 1% THD medium. Three doses of T3 were used: 1 nM (just below the minimum for normal T3 levels, 1.54 nM, in the hypothyroid range), 3 nM (around the maximum for normal T3 levels, 3.08 nM), and 10 nM (in the hyperthyroid range). As shown in Figure 3A, T3 significantly increased neurite outgrowth, in a dose-dependent manner. Maximum levels (10.83%) were obtained in the presence of 10 nM T3 (Figure 3A). In time course studies using 3 nM T3 (Fig. 3B), maximum (significant, p<0.05) levels (8.36%) were seen after 72 h of incubation.

**Figure 4:**
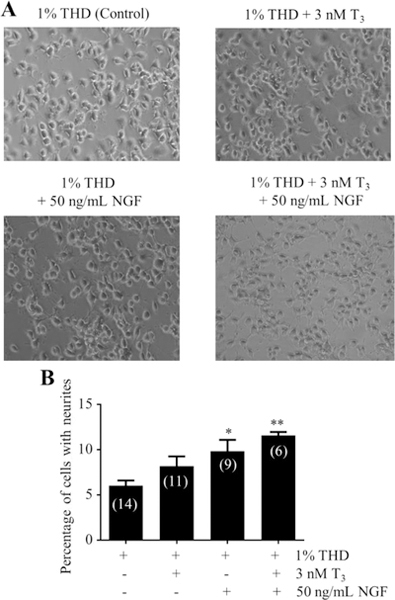
Effects of T3 and NGF on neurite outgrowth in NS-1 cells. Cells were incubated in 1% THD medium in the presence of 3 nM of T3, and 50 ng/mL NGF for 72 h and assayed for neurite outgrowth. (A) Typical images of cells exposed to the different treatments. These experiments were repeated at least three times. (B) Data are presented as a percentage mean of cells with neurites ± SEM. “*” (p<0.05) and “**” (p<0.01) were obtained by comparing to the 1% THD (control) group using ANOVA followed by Student–Newman–Keuls multiple comparison tests. Numbers within parenthesis inside each bar correspond to the replicates (“n”) for each group.

To determine whether T3 could enhance neurite outgrowth in the presence of NGF, we tested the effect of T3 and NGF on the neurite outgrowth of NS-1 cells. For this, cells were grown in 1% THD medium supplemented with 50 ng/mL NGF ± 3 nM T3 for 72 h. Figure 4A illustrates typical microscope images of the cells in the different treatments.

As shown in Figure 4B, T3 treatment for 72 h enhanced neurite outgrowth from 6.04% to 8.19% in the absence of NGF (1.36-fold) and from 9.83% to 11.59% in the presence of NGF (1.18-fold). Thus, the effects of T3 and NGF on neurite outgrowth in NS-1 cells were additive.

**Figure 5:**
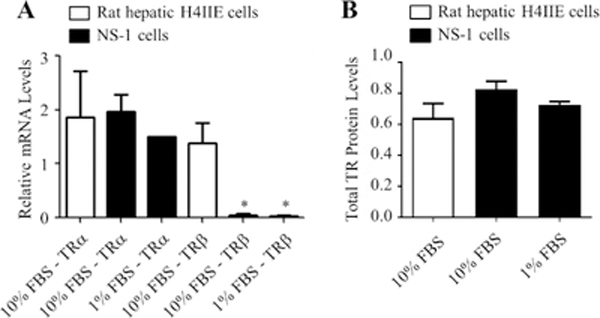
Expression of thyroid hormone receptors (TRs) in NS-1 and hepatic H4IIE cells. Cells were incubated in 10% or 1% FBS medium for 72 h. (A) RNA was isolated, reverse-transcribed and analyzed using quantitative real-time polymerase chain reaction (qRT-PCR). Data are presented as relative mRNA mean level ± SEM for n=3 for all the conditions. “*” (p<0.05) was obtained by comparing to the H4IIE-10% FBS-TRβ using ANOVA followed by Student–Newman–Keuls multiple comparison tests. (B) Protein levels were quantitated using in-cell Western assays. Data are presented as relative protein mean levels ± SEM. This experiment was performed in sextuplicate and repeated 3 times.

To examine whether these cells express any TR that could mediate the effects of T3 on their neurite outgrowth, mRNA, and protein analyses, we performed qRT-PCR and in-cell Western blotting studies, respectively. Rat liver cells were used as the control for these studies. As shown in Figure 5A, NS-1 cells expressed detectable TRα and TRβ mRNA levels. Interestingly, TRβ mRNA expression was 25 times lower (p<0.05) in the NS-1 cells than in the rat liver control samples (Figure 5A). In the case of TRα, the mRNA expression in NS-1 cells was comparable to the expression in the rat liver control sample (Figure 5A). Total TR protein expression levels were similar in both cell lines (Figure 5B). These data suggest that either of these receptors, but most likely TRα, could mediate the effects of T3 in the NS-1 cells.

**Figure 6:**
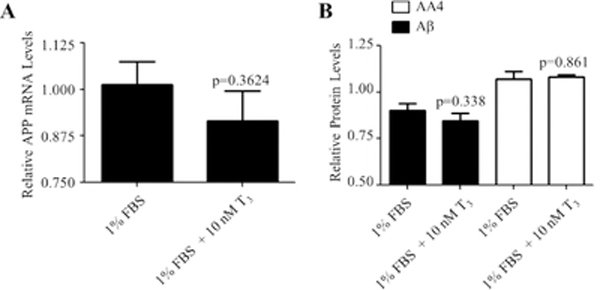
Effects of T_3_ on the expression of AA4 and Aβ in 1% FBS. Cells were incubated in the indicated medium in the presence of 10 nM of T_3_ for 72 h. (A) Total RNA was isolated, reverse transcribed and subjected to qRT-PCR. Data are presented as relative mRNA mean levels ± SEM for n=13. The p-value was obtained using the Student’s t-test. (B) Protein levels were quantitated using in-cell Western analysis. Data are presented as relative protein mean levels ± SEM for n=4. The p-values were achieved by comparing to 1% FBS using ANOVA followed by Student–Newman–Keuls multiple comparison tests.

Subsequently, the expression levels of APP and Aβ in response to 10 nM T3 were examined. As shown in Figure 6A, there was a small reduction (9.5%) in APP mRNA levels in response to T3, but APP protein expression was unaffected by T3 treatment. The Aβ protein expression was also unaltered in response to T3 (Figure 6B). The expression levels of tau in response to T3 were also examined.

**Figure 7:**
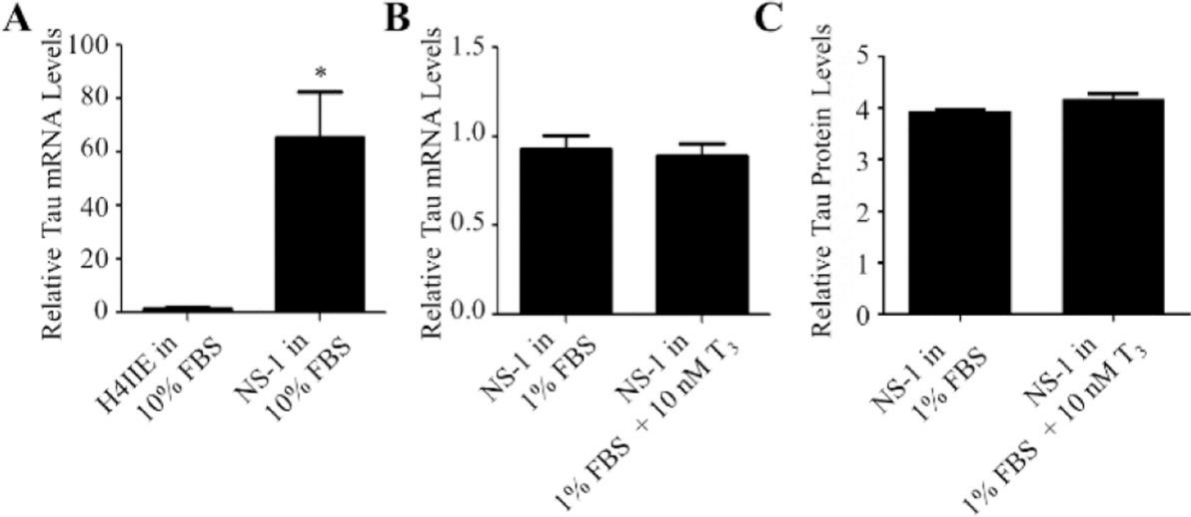
Effects of T3 on the expression of tau. (A) H4IIE and NS-1 cells were incubated in 10% FBS medium for 72 h. Total RNA was isolated, reverse transcribed, and subjected to qRT-PCR. “*” (p<0.05) was obtained by comparing to H4IIE using the Student’s t-test. This experiment was performed in triplicate and repeated three times. (B) NS-1 cells were incubated in 1% FBS medium alone or the presence of 10 nM of T3 for 72 h. The analysis of the RNA samples was done as described in (A). The data are presented as relative mRNA mean levels ± SEM for n=8. (C) NS-1 cells were incubated in 1% FBS medium alone or the presence of 10 nM T3 for 72 h. Protein levels were quantitated using in-cell Western analysis. The data are presented as relative protein mean levels ± SEM for n=4.

The expression of Tau mRNA was 44.3-fold higher in the NS-1 than in the liver cells (Figure 7A). However, the tau mRNA expression in NS-1 cells was not increased by T3 treatment (Figure 7B). The protein expression of tau was only slightly increased (1.06-fold) in response to T3 (Figure 7C).

Tau has been shown to be regulated by alternative splicing of exon 10 [32,33]. The exon 10 of tau encodes the second of four imperfect microtubules (MT)-binding repeats in the C-terminal region of the tau protein [33].

**Figure 8:**
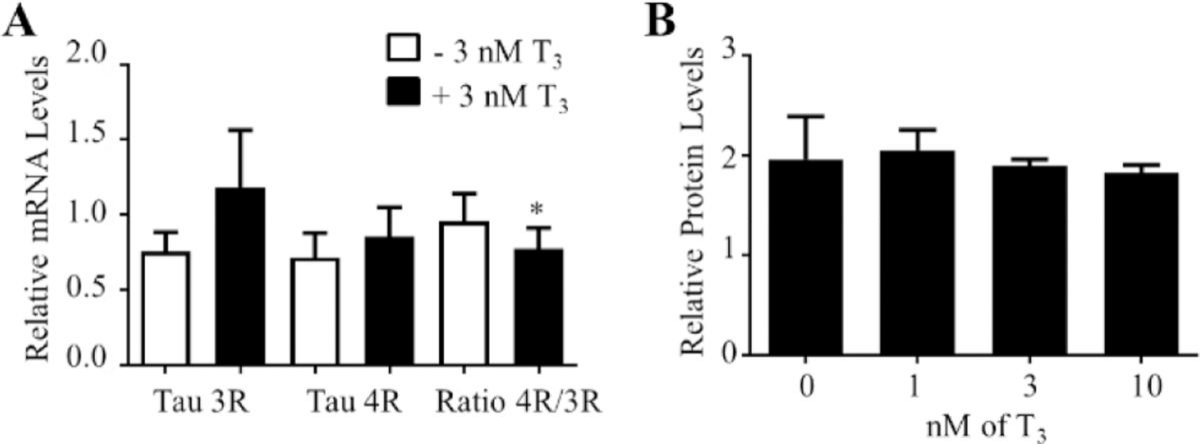
Effects of T3 on the splicing of tau exon 10. (A) Total RNA was prepared from cells treated with 1% THD ± 3 nM T3 and analyzed as described above. The data are presented as relative mRNA mean levels ± SEM for n=8. “*” (p<0.05) was obtained using ANOVA followed by Student–Newman–Keuls multiple comparison tests. (B) In-cell Western analysis of tau protein in cells treated with increasing amounts of T3 and 1% THD. The data are presented as relative protein mean levels ± SEM for n=21.

Tau isoforms not including exon 10 have three MT-binding domains (3R), whereas tau isoforms including exon 10 have four MT-binding domains (4R) [33,34]. It has been documented that changes in its isoform ratio, due to the regulation of splicing, can lead to AD [32]. Interestingly, some exon 10 mutations are missense that influences MT-binding [32]. However, the majority are silent at the protein level but alters the ratio of Tau 4R and Tau 3R [32]. Thus, the following experiment was to determine if T3 treatment alters the splicing of tau exon 10 in NS-1 cells. Primers specific for each tau isoform (3R or 4R) were designed and used in qRT-PCR. For this experiment, the NS-1 cells were grown in 1% THD medium and treated with or without 3 nM T3 for 72 h. As shown in Figure 8A, T3 treatment increased the levels of Tau 3R and Tau 4R by 1.6 and 1.2-fold, respectively. The ratio of Tau 4R/3R was significantly reduced (19.5%) when treated with T3. Similar to using 1% FBS, T3 treatment did not affect tau protein expression in the presence of 1% THD medium (Figure 8B).

## Discussion

Herein, it was demonstrated that in 1% THD medium, T3 treatment enhanced neurite outgrowth in NS-1 cells in a time- and dose-dependent manner. Adding NGF increased the effects of T3 even more. qRT-PCR and in-cell Western analyses confirmed that the NS-1 cells express detectable TRα and some TRβ. These data suggest that either of these receptors, but most likely TRα, could mediate the effects of T3 in the NS-1 cells. Although the overall expression of tau protein in NS-1 cells was not induced upon T3 treatment, the hormone did result in a change in the relative levels of tau 3R and 4R. Thus, it might be possible that tau contributes to the T3-dependent induction of neurite outgrowth observed in NS-1 cells.

Evidence obtained from these studies in conjunction with previous studies suggests that there is an apparent link between AD and hypothyroidism. However, it is not clear what mechanisms are impaired that lead to this outcome. One possible explanation is the finding that thyroid hormone down-regulates the expression of the APP gene [35–37]. This is a critical and significant finding considering that the accumulation of Aβ results from high expression levels of APP [38,39]. Thus, having normal levels of thyroid hormone help maintain APP levels to a minimum and subsequently preventing Aβ overproduction. Surprisingly, two independent studies showed an association between the presence of the apo E4 allele and low thyroid hormone levels [40,41] suggesting that T3 also plays a critical role controlling the removal of Aβ. Interestingly, T3 appears to regulate the splicing and maturation of the tau mRNA, as previously shown [42,43] and herein. Independent of these findings, further studies are required to confirm the role of T3 levels in AD development.

NGF, one of the substances used in our studies, is known to be essential for the growth, maintenance, and survival of nerve cells [44]. NGF is synthesized as a precursor molecule (proNGF) that requires proteolytic cleavage before it can be considered a biologically active protein [44]. Once it is cleaved, NGF binds either to its high-affinity TrkA receptor or to the low-affinity p75NTR receptor [45,46]. Binding of NGF to the TrkA receptor is what stimulates signal transduction pathways mediating most of the survival and growth effects of NGF, including neurite outgrowth [46]. Binding of NGF to the p75NTR receptor mainly leads to a positive modulation of the NGF/TrkA binding, but p75NTR activation could also cause apoptosis depending on the type of protein interacting with the receptor [46–52].

ProNGF is also able to interact with both receptors, but those interactions have been partially linked to the neuron dysfunction seen in AD [45]. In fact, it has been demonstrated that there a direct correlation between increased proNGF levels and decreased cognitive performance in subjects diagnosed with mild AD [53]. The binding of proNGF to the TrkA receptor promotes neuronal survival and neurite outgrowth similar to mature NGF, but proNGF is about five times less active than mature NGF [54,55].

Also, proNGF tends to be utilized less efficiently than mature NGF resulting in a higher accumulation of proNGF in neurons [56]. Once at high levels, proNGF can interact with the p75NTR receptor resulting in apoptosis [57].

Another consequence of elevated levels of proNGF in the brain is related to the finding that TrkA inhibits, and p75NTR activates the activity of β-secretase [58]. Thus, activation of the p75NTR receptor by an excess of proNGF most likely results in increased levels of Aβ leading to the formation of Aβ plaques [58]. Interestingly, it has been shown that hypothyroidism not only impairs the maturation of NGF but also enhances the expression and proteolysis of the p75NTR receptor suggesting increased signalling via this receptor [59].

Furthermore, studies in hypothyroid rats demonstrated that the lack of thyroid hormone enhances the expression levels of Aβ peptides, especially Aβ−42 which is more prone to aggregate, in correlation with an increase in β-secretase activity [60].

**Figure 9:**
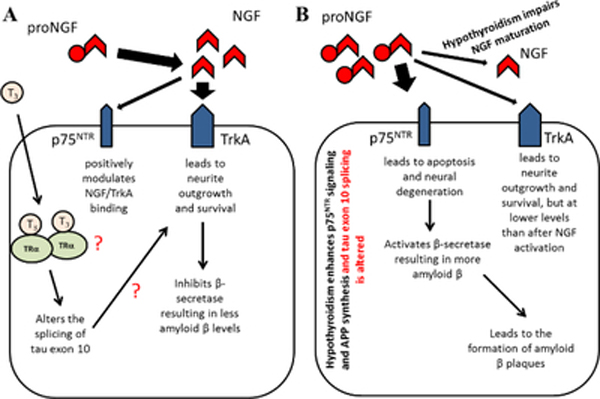
The hypothetical link between hypothyroidism, NGF maturation and function, APP synthesis and processing, and tau protein expression. (A) In the presence of T3, proNGF gets converted to its active form, NGF, which mainly activates the TrkA receptor. Activation of the TrkA receptor by NGF leads to neurite outgrowth and survival as well as inhibition of β-secretase resulting in lower Aβ levels. Also, T3 leads to activation of the TRα receptor which in turns reduces APP transcription and alters the splicing of tau exon 10. It is possible that changes in the ratio of tau 4R/3R contribute to neurite outgrowth and survival promoted by activation of the TrkA receptor by NGF. (B) In the hypothyroid condition, NGF synthesis is impaired preventing the positive effects of activating the TrkA receptor. Instead, the high levels of proNGF enhance signalling via the p75NTR receptor which leads to apoptosis and neural degeneration. The lack of T3 also leads to an increase in transcription of APP and changes in tau exon 10 splicing. Furthermore, the activity of β-secretase is no longer inhibited. Consequently, the Aβ levels increase resulting in the formation of Aβ plaques.

It is also important to mention that thyroid hormone not only has been shown to control the processing of APP but its synthesis [36,61]. Thyroid hormones via their nuclear receptors down-regulate the transcription of the APP gene [36]. Thus, hypothyroidism leads to an overproduction of APP, which is now available to be processed by the increased activity of β-secretase. These suggest a possible link between hypothyroidism, increased APP synthesis, and processing, as well as decreased NGF maturation and function (illustrated in Figure 9). It is also possible that hypothyroidism-impaired NGF maturation results in elevated levels of TR that overcompensate and propel the cell towards survival.

Although differential expression levels of AA4, Aβ, and tau proteins were not detected in NS-1 cells in response to treatments with T3, we found that the mRNA expression levels of mainly tau 3R increased upon treatment with T3.

However, it is unclear if tau 3R is further involved in a hypothyroidism-dependent manner with a potentially increased risk of AD and other neurodegenerative diseases. Increases in tau 3R have been associated with neuronal proliferation and survival which correlate with the effects of T3 on the NS-1 cells.

In summary, these studies suggest that T3 treatment enhances neurite outgrowth of NS-1 cells and further supports the possibility of the involvement of this hormone in the prevention of AD. Similarly, we observed potentiation of neurite outgrowth in T3-treated, NGF-treated NS-1cells in comparison to NS-1 cells treated with T3 alone. These T3-dependent effects on neurite outgrowth appeared to be mainly time- and dose-dependent. The NS-1 cells expressed high levels of the mRNAs for TRα and some of the TRβ isoform, either of which could be responsible for T3-dependent effects in neurite outgrowth.

This information is critical to better understand the link between hypothyroidism and AD as well as the role of thyroid hormone in brain development. Also, this information could provide a basis for future prevention and treatment of AD, especially in hypothyroid patients.
